# *RBM20* p.Arg636Cys: A Pathogenic Variant Identified in a Family with Several Cases of Unexpected Sudden Deaths

**DOI:** 10.3390/jcm14030743

**Published:** 2025-01-24

**Authors:** Rebeca Lorca, Alberto Alén, María Salgado, Rosario Misiego-Margareto, Javier Dolado-Cuello, Juan Gómez, Vanesa Alonso, Eliecer Coto, Pablo Avanzas, Antonia Martínez-Hernández, María Paz Suárez Mier

**Affiliations:** 1 Área del Corazón, Hospital Universitario Central Asturias, 33011 Oviedo, Spain; 2 Unidad de Cardiopatías Familiares, Área del Corazón y Departamento de Genética Molecular, Hospital Universitario Central Asturias, 33011 Oviedo, Spain; 3 Departamento de Biología Funcional, Universidad de Oviedo, 33003 Oviedo, Spain; 4 Instituto de Investigación Sanitaria del Principado de Asturias, ISPA, 33011 Oviedo, Spain; 5 Redes de Investigación Cooperativa Orientadas a Resultados en Salud (RICORs), 28029 Madrid, Spain; 6 Instituto de Medicina Legal y Ciencias Forenses de Asturias, 33011 Oviedo, Spain; 7 Departamento de Medicina, Universidad de Oviedo, 33003 Oviedo, Spain; 8 Centro de Investigación Biomédica en Red de Enfermedades Cardiovasculares (CIBERCV), 28029 Madrid, Spain; 9 Histopathology Service, National Institute of Toxicology and Forensic Sciences, 28232 Madrid, Spain; mariapaz.suarez@justicia.es

**Keywords:** dilated cardiomyopathy (DCM), sudden cardiac death (SCD), RBM20, genetic testing, cardiovascular prevention

## Abstract

**Background**: Dilated cardiomyopathy (DCM) can be an inherited condition related to premature sudden cardiac death (SCD). Pathogenic variants in some genes, like *LMNA*, *SCN5A*, *FLNC* or *RBM20*, have been linked to an increased risk of SCD. Although genetic study can help to stratify the arrhythmic risk, there are no specific guidelines for *RBM20* carriers’ management. We aimed to evaluate the genetic profile and clinical features of all DCM patients with pathogenic variants in *RBM20.* **Methods**: We identified all carriers of pathogenic variants in *RBM20* in a single national center that specializes in inherited cardiac conditions. Forensic and molecular autopsies provided crucial information. **Results**: We identified a large family with inherited DCM due to *RBM20* p.Arg636Cy and several SCDs. The proband was a 37-year-old male who suffered an unexpected SCD despite presenting a mild DCM phenotype with normal left ventricular ejection fraction. Family screening identified four other carriers, who were asymptomatic, but presented concealed mild DCM phenotypes. Family history revealed that six other relatives (two of them obligate carriers) had also suffered sudden deaths at young ages. **Conclusions**: We present an informative family with DCM, due to *RBM20* p.Arg636Cys, and high rates of SCD, even in members with mild DCM phenotypes. ICD implantation to prevent SCD should be carefully evaluated in all *RBM20* p.Arg636Cys carriers. Moreover, the frequent development of AF and HF progression requires specific awareness.

## 1. Introduction

Sudden cardiac death (SCD) is the most severe manifestation of cardiovascular disease and can result from an inherited cardiovascular condition. Therefore, achieving a definitive diagnosis is of critical importance, especially in young individuals [1]. In fact, cardiomyopathies are recognized as the primary cardiac conditions associated with arrhythmogenic syndromes responsible for SCD in the young [2]. In this sense, forensic or clinical autopsies, including postmortem genetic testing, could provide crucial information about the underlying cause of SCD and help identify at-risk relatives [3,4,5]. However, malignant electrical manifestations such as SCD may sometimes occur at very early stages, even before the development of evident structural heart defects [6].

Primary dilated cardiomyopathy (DCM) is a cardiomyopathy characterized by either left ventricular or biventricular systolic dysfunction and dilation that cannot be solely explained by abnormal loading conditions or coronary artery disease [7,8]. Although its clinical course can be variable, DCM is considered one of the leading causes of death, heart failure (HF) and heart transplantation [9]. In this context, arrhythmias can not only be triggered by DCM itself but also cause or exacerbate DCM and HF [10].

Familial DCM, an inherited form of the condition, is typically passed from parents to their children following an autosomal dominant inheritance pattern. The management of inherited DCM may involve lifestyle modifications, HF-related treatment and close monitoring to prevent malignant cardiac arrhythmias [11,12]. The risk of SCD varies depending on numerous clinical factors such as the patient’s age, gender, the severity of the condition and the presence of other cardiac abnormalities [11,12]. In some cases, an implantable cardioverter-defibrillator (ICD) may be recommended. Early diagnosis and appropriate management are critical to reducing the risk of SCD in all patients with inherited DCM. Nonetheless, accurate risk stratification remains a significant challenge, even in cases with significant ventricular dysfunction. For instance, the DANISH clinical trial demonstrated no mortality benefit from ICD implantation for primary prevention in patients with non-ischemic DCM [13]. Conversely, it is recognized that patients with inherited DCM have a higher risk of SCD compared to those with non-inherited DCM [11,12]. In this regard, genetic information can aid in stratifying arrhythmic risk. Indeed, several genes have been linked to an increased risk of SCD [11,14]. Genes associated with higher arrhythmic risk include genes coding for the nuclear envelope (*LMNA*, *EMD* and *TMEM43*), desmosomal (*DSP*, *DSG2*, *DSC2* and *PKP2*) or certain cytoskeletal proteins and *FLNC* [15,16]. Pathogenic variants in the *DES* gene are associated with a wide spectrum of cardiomyopathy phenotypes [16]. Some of these variants have been documented in families with a high prevalence of arrhythmias and SCD. [17]. Notably, a dedicated risk score for life-threatening ventricular tachyarrhythmias has been developed for laminopathies [18] and phospholamban (*PLN*) variant carriers [19]. Therefore, the ESC guidelines advocate for a more personalized risk stratification approach that is based on genotype information. Consequently, genetic counseling may be recommended for affected individuals and their families to provide information on the inheritance pattern, SCD risk and family planning options [11,12]. DCM patients harboring DCM-causing variants in high-risk genes (e.g., LMNA, *EMD*, *FLNC*, *PLN*, *DSP* or *TMEM43*) should be considered as high-risk patients for SCD, and primary prevention ICD implantation should be considered when left ventricular ejection fraction (LVEF) thresholds are higher than 35% [16,20].

Recently, *RBM20* (OMIM: 613171), a gene encoding RNA-binding motif protein 20, has also been identified as a high-risk gene associated with ventricular arrhythmias [14]. It is also considered as a high-risk genetic profile for SCD risk stratification in recent ESC guidelines [16,20]. However, the specific risk factors for SCD related to this gene are not yet well established. Therefore, the risk stratification of SCD in *RBM20* carriers is an active area of research.

In this context, we aimed to evaluate the genetic profile and clinical features of patients with inherited DCM due to a pathogenic variant in *RBM20*.

## 2. Materials and Methods

### 2.1. Patients’ Population

In this retrospective study, we reviewed all consecutive patients referred for genetic testing with an inherited cardiomyopathy diagnosis from 2018 to 2023 at a national reference center for inherited cardiovascular conditions. Only families with pathogenic variants in *RMB20* were included in this study. We retrospectively collected clinical data from this cohort. We reviewed their birth data, genetic data and clinical data, including personal and family history, cardiovascular (CV) symptoms, CV risk factors (CVRF), electrocardiogram (ECG), transthoracic echocardiogram (TTE), cardiac magnetic resonance (CMRI), Holter monitoring, device implantation and postmortem cardiac findings, if available.

All patients had signed a written consent to grant access to their genetic data for investigational purposes. The research protocol followed institutional ethics guidelines. This study was evaluated by the local Ethical Committee (CEImPA 2022.254).

### 2.2. Genetic Testing

Genetic testing was performed on all patients who accepted to undergo genetic testing and signed the inform consent. If the patient was deceased, a first-degree relative (e.g., parents or legal partner) provided consent. DNA was extracted from peripheral blood leukocytes using the standard salting-out method [21]. In cases where a deceased patient’s blood sample was unavailable or degraded, DNA was extracted from preserved tissue. Index cases underwent sequencing using an NGS cardiovascular panel which included over 200 genes related to CV disease, including DCM. Technique details of this methodology are described in [22,23,24,25,26,27]. Variants were interpreted according to the American College of Medical Genetics and Genomics (ACMG-AMP) 2015 Standards and Guidelines [28]. Based on the ACMG-AMP criteria, variants were classified as pathogenic variants (PVs), likely pathogenic variants (LPVs) and variants of uncertain significance (VUS). Likely benign or benign variants were not reported. If a PV or LPV was identified, its presence was confirmed via direct Sanger sequencing. Genetic family screening was also perfumed using Sanger sequencing.

### 2.3. Statistical Analysis

Statistical analyses were conducted using SPSS v.19. Continuous variables are presented as mean ± SD, while categorical variables are summarized as frequencies or percentages. The Chi-square test or Fisher’s exact test was used to compare categorical data, with *p* < 0.05 considered statistically significant.

## 3. Results

**Figure 1:**
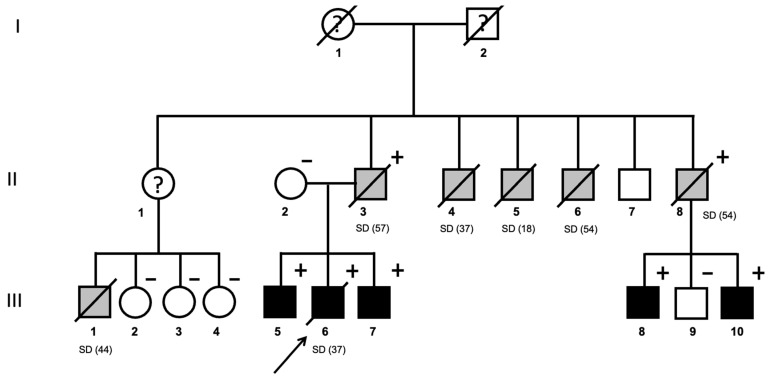
*RBM20* p.Arg636Cys carriers: family pedigree. SD, sudden death. Age of SD in brackets. Symbols denote sex and disease status: +, carriers; −, noncarriers; without a sign, genetic status unknown; box, male; circle, female; black darkened, DCM phenotype; gray darkened, unexpected SD; symbol clear, negative phenotype; ?, unknown phenotype; slashed, deceased; arrow, proband.

From the consecutive patients referred for genetic testing due to DCM, we identified one family (Figure 1) harboring the pathogenic variant c.1906C>T, p.Arg636Cys in *RBM20* (NM_001134363.3) [29].

**Figure 2:**
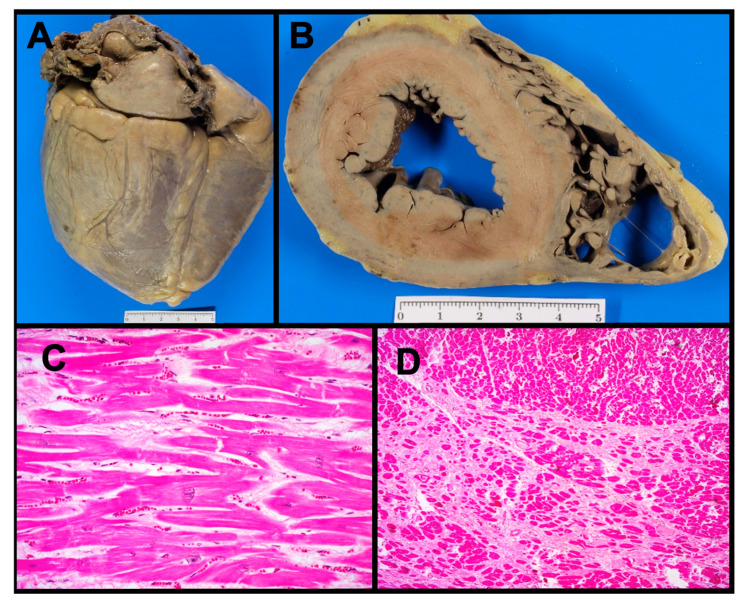
Pathological findings of patient III.6’s heart examination, carrier of the pathogenic *RBM20* p.Arg636Cys variant. (**A**) Posterior/inferior view of the external aspect of the heart. (**B**) Transversal biventricular section. (**C**) Microscopy image of the left ventricular wall, with cardiomyocytes slightly hypertrophic without disarray (HE staining, 20×). (**D**) Mild subendocardial interstitial fibrosis (HE staining, 10×).

Patient III.6 was a 37-year-old man who suffered from SCD while working (Figure 1). At the age of 35, he had been referred to a cardiologist after experiencing syncope in the context of rapid atrial fibrillation (AF). Upon further evaluation, the patient had spontaneously recovered sinus rhythm. Moreover, 72 h ECG monitoring showed no additional arrhythmias. Baseline ECG in sinus rhythm and the initial TTE evaluation were described as normal, except for a mild left ventricular (LV) dilation in the context of the recent rapid atrial fibrillation. LVEF was normal. In follow-up visits, 6 months later, without cardiological treatment, patient III.6 remained in sinus rhythm, with normal LVEF, and recovered with LV normal volumes (within the upper normal limit). In summary, he had presented a single episode of syncope, in the context of rapid AF, with normal cardiological findings on control studies. Unfortunately, later on, he suffered unexpected SCD while working. Postmortem cardiac examination revealed a heart weight of 486 g (>p95) with mild LV dilatation (3.6 cm diameter) (Figure 2). Further analysis found hypertrophic cardiomyocytes but without disarray (Figure 2C). Additionally, mild focal subendocardial interstitial fibrosis was observed under microscopic examination (Figure 2D). Despite this mild DCM phenotype, with only mild LV dilatation and focal fibrosis, a possible underlying inherited cardiomyopathy as the primary cause of the SCD was suspected. Therefore, a blood sample was collected and stored for further eventual genetic analysis.

Subsequently, first-degree relatives were advised to undergo cardiological evaluation. The family pedigree was evaluated (Figure 1), revealing a striking number of sudden deaths, all occurring without prior clinical warning symptoms. Initially, patient II.2 (his mother) and patient III.5 (older brother) were given an appointment at the cardiology clinic.

Patient II.2, as the legal guardian, signed the inform consent to perform the molecular autopsy of patient III.6. As a result, the legal process to access biological samples (stored at the National Institute of Toxicology and Forensic Science) was initiated. Patient III.5, during the first evaluation, underwent a TTE exam that revealed a mild LV dilation. Given the family history of SD (Table 1) and the potential delay in the molecular autopsy legal process, before waiting for the biological samples of his brother to arrive, genetic testing was also offered to patient II.5. Consequently, both patients were almost simultaneously sequenced using the same NGS cardiovascular panel. In the meantime, CMRI for patient III.5 was scheduled.

Genetic results showed that they were both carriers of the *RBM20* p.Arg636Cys variant. Based on the ACGM criteria [28], this variant was classified as pathogenic. As a result, cascade genetic testing was performed. CMRI confirmed that patient III.5 had concealed DCM.

Family history revealed that this family had experienced six additional unexpected SD before age 60, with no known prior history of cardiovascular disease or warning symptoms. Moreover, two of the relatives who had suffered SD were obligate carriers of the pathogenic *RBM20* p.Arg636Cys variant. A summary of these relatives is presented in Table 1.

Genetic screening was encouraged for all relatives, including patient II.1. Unfortunately, patient II.1 refused to undergo genetic testing. However, given the sudden death of patient III.1, we emphasized the importance of evaluating this part of the family. Fortunately, we could rule out that neither patient III.2, III.3 nor III.4 were carriers of the pathogenic *RBM20* variant (Figure 1). On the other hand, thanks to genetic family screening, in 2020, three additional carriers of the variant were identified: patients III.7, III.8 and III.10 (Figure 1). The baseline cariological phenotype evaluation of all identified carriers is summarized in Table 2. Patient III.7 underwent AF ablation in May 2024, and LVEF in control TTE from 2024 dropped to 39%.

**Figure 3:**
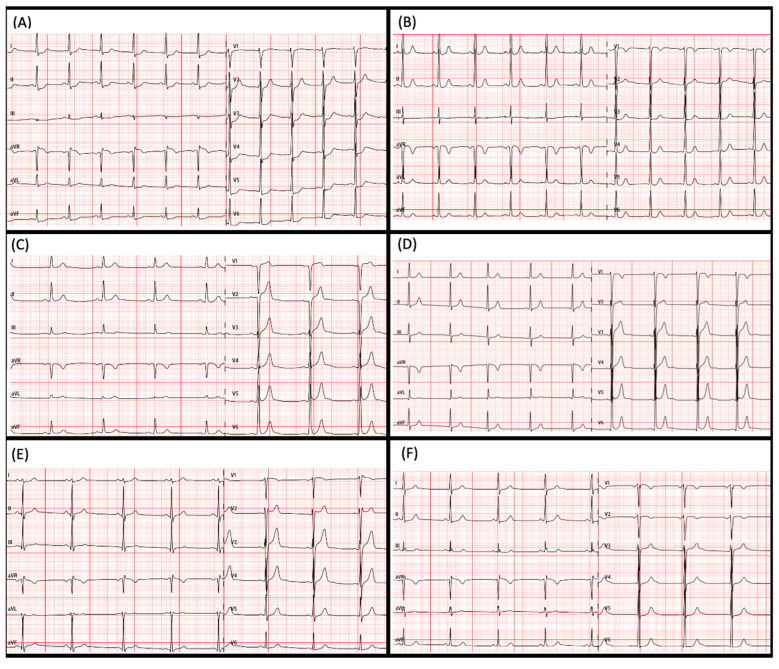
Electrocardiograms (ECGs). (**A**) Patient III.5, a carrier of *RMB20* p.Arg636Cys pathogenic variant with subclinical dilated cardiomyopathy (DCM); (**B**) Patient III.6, who suffered a sudden cardiac death with mild DCM prior to genetic analysis (*RBM20* p.Arg636Cys carrier); (**C**) Patient III.7, a subclinical DCM variant carrier; (**D**) Patient III.8, a subclinical DCM variant carrier. (**E**) Patient III.9, a non-carrier; (**F**) Patient III.10, a subclinical DCM variant carrier.

Basal ECG was unable to distinguish those carriers with a positive phenotype (Figure 3) from non-carriers. All *RBM20* p.Arg636Cys carriers, except patient III.5 (Figure 3A), had normal ECGs, including patient III.6 (Figure 3B). In fact, ECGs from two brothers, one being an affected carrier (patient III.8) and the other a healthy non-carrier (patient III.9), were nearly identical (Figure 3D,E).

Conversely, cardiac imaging techniques revealed that all carriers presented subclinical mild DCM phenotypes (Table 2, Figure S1). As previously mentioned, patient III.6 suffered SCD without prior detection of any ventricular arrhythmias. Like his brother, in patient III.5, no ventricular arrhythmias were detected, and AF was the only arrhythmia registered. However, in patient III.7, baseline Holter monitoring did identify a non-sustained ventricular tachycardia. Furthermore, patient III.7 underwent AF ablation in May 2024 (Table 2). ICD indication was carefully discussed by the heart team, and ultimately, all carriers were considered eligible for ICD implantation for primary SCD prevention. Patients III.5, III.7, III.8 and III.10 engaged in shared decision-making, and all patients decided to undergo ICD implantation.

## 4. Discussion

Currently, identifying DCM patients with high arrhythmogenic risk remains a real clinical challenge. The current European HF guidelines recommend ICD implantation for primary prevention in DCM patients with reduced LVEF (below 35%) and symptomatic HF [11,12]. However, since the release of clinical data from the DANISH trial [13], the benefit of ICD in non-ischemic DCM for primary prevention has been questioned [14]. Recent results from the extended follow-up of the DANISH trial showed that ICD implantation did not result in an overall survival benefit in patients with non-ischemic DCM with reduced LVEF, regardless of the modified Heart Failure Collaboratory (mHFC) score [30]. However, the initial trial did show that the younger the patient, the higher the potential benefit seemed to be [13]. On the other hand, the underlying etiology of DCM beyond the label of “non-ischemic” should be carefully outweighed for SCD risk stratification [11,12]. In fact, we believe that patients with inherited DCM, including those with the worst arrhythmogenic profiles, may not even be represented in the trials’ cohort [13]. Malignant ventricular arrhythmias may occur at mild or moderate LVEF reductions [31]. Furthermore, SCD can be the very first manifestation of the disease in some inheritable forms of DCM and may even precede the clinical onset of DCM. In this regard, the current ESC guidelines provide a specific warning for genes with concerning arrhythmogenic profiles [11,12]. ICD should be considered in DCM patients with an LVEF < 50% and ≥2 risk factors, including syncope, late gadolinium enhancement, inducible sustained monomorphic ventricular tachycardia at programmed electrical stimulation and pathogenic variants in the *LMNA*, *PLN*, *FLNC* and *RBM20* genes [11]. In this sense, pathogenic *RBM20* variants have been associated with particularly aggressive phenotypes, specifically the early onset of the disease, malignant arrhythmias and high premature mortality [32,33,34,35,36]. However, the major concern in *RBM20* gene alterations is that severe arrhythmogenic phenotypes can present even with a normal LVEF, as seen in the patient we presented in this study. Therefore, SCD risk stratification in individuals with DCM due to *RBM20* pathogenic variants remains an area of ongoing research. Currently, there are no specific established guidelines for SCD risk stratification in *RBM20* carriers. It remains unclear how much the pathogenic variants specifically contribute to the overall SCD risk and when exactly to implant the ICD in primary prevention. Recently, a longitudinal follow-up for 143 *RBM20* variant carriers reported that LVEF impairment and the *RBM20* p.Arg634Gln variant conferred additional risk [36].

To date, most pathogenic *RBM20* reported variants are due to *missense* pathogenic variants, and most variants are located in a hotspot arginine–serine domain [33,34]. An elegant review summarized that from all rare variants reported, and only 13 of them could be considered as a disease-causing variant (LP or L) [37]. It was reported that disease-associated variants in *RBM20* may lead to aberrant splicing through different mechanisms that may be dependent on the localization [38]. Most reported LP variants were located in the hot-spot region from exon 9 (from amino acids 634 to 638). However, many of these LP variants could not be classified as definitely P due to a lack of additional data [33,39], including *RBM20* p.Arg636Cys [37]. The *RBM20* p.Arg636Cys variant’s first submission to ClinVar dates back to 2013 (ClinVar Variant ID# 43980). At the time of this very fist submission, last evaluated in 2017, it was classified as a variant of unknown significance according to the ACMG/AMP criteria applied by the submitters (PS4_Supporting, PM1, PM5). In the literature, the *RBM20* p.Arg636Cys variant had be described 3 years before, in 2010, by Li et al. [34]. Available clinical data of all relatives reported in the literature are summarized in Table 3 [34]. The proband initiated cardiological evaluation due to family history. At first evaluation, she was an asymptomatic 43-year-old women with subclinical DCM. Genetic testing identified that she was a carrier of the variant *RBM20* p.Arg636Cys. As this paper was published prior to the ACMG/AMP classification criteria, the variant was reported as “mutation”. Her sister died at the age of 25, with a prior history of peripartum cardiomyopathy. Moreover, her two nephews underwent heart transplant at the age of 14 and 16 years old. Genetic information about these three deceased relatives to strongly support the segregation and pathogenicity of *RBM20* p.Arg636Cys was, unfortunately, unavailable. Her son was also an asymptomatic carrier with subclinical DCM. No additional relatives were reported [34], and to the best of our knowledge, after Li et al., no other families have been described in the literature [34]. Cannie et al. also reported that 7.4% of their cohort harbored *RBM20* p.Arg636Cys, but no specifical clinical data regarding carriers were reported [36].

In a recent review, Jordà P et al. [37] performed a comprehensive update of rare variants in *RBM20* and classified the protein change p.(Arg636Cys) as LP. Moreover, in ClinVar database (www.ncbi.nlm.nih.gov/clinvar/intro (accessed on 1 January 2025)), four additional submissions did classify this variant as pathogenic. Therefore, global interpretation for the variant was considered “conflicting” [36]. Until recently, that VUS submission was flagged as “submission do not contribute to the aggregate classification or review status for the variant”. Other *missense* changes affecting the same residue, reported at ClinVar, but without conflicting interpretations of their pathogenicity, are summarized in Table 4. Another single nucleotide change at the same position, but resulting in a different amino acid, had already been reported as pathogenic (c.1906C>A, p.Arg636Ser). Moreover, another change in the next residue, but also affecting the same amino acid (c.1907G>A, p.Arg636His), was also reported as LP [33,34,39,40]. Furthermore, there are functional studies supporting the pathogenicity of another change, *RBM20* c.1907G>T, p.Arg636Leu, showing both in cell culture and in human cardiac tissue, and the protein was abnormally located at the cytoplasm [38]. In addition, a synonymous change at the same amino acid was also described. Moreover, functional studies support its pathogenicity. It was demonstrated that whereas the wild-type form of *RBM20* was localized in the nuclei, *RBM20* p.Arg636Cys was retained in the cytoplasm, revealing a severe mislocalization in vitro [41]. Finally, a recent publication by Cannie et al. [36] also labeled it as LP (fulfilling the PM1, PM2, PM5, PP3 and PP5 criteria). Consequently, we believe that sharing the segregation information provided in this study is important to classifying *RBM20* p.Arg636Cys as definitely pathogenic.

On the other hand, we consider the high-risk presentation of this family to be equally worth sharing. In this family, seven relatives suffered unexpected SD before age 60. In fact, in the absence of the genetic information, patient III.6 suffered a clinically unpredictable SCD. He presented a normal LVEF and a mild DCM phenotype, which were confirmed using the gold standard diagnostic technic: the anatomopathological exam. These findings are consistent with other groups’ concerns as SCD can be the first manifestation of the disease (before cardiology contact) or present with mild cardiological findings whether on ECG, Holter or imaging studies. It is known that all carriers of *RBM20* pathogenic variants should receive close cardiological monitoring, genetic counseling and ICD in primary prevention; they should be carefully outweighed in all clinical visits. Moreover, as already proposed for certain *RBM20* pathogenic variants [36,42], we believe that the threshold for ICD implantation should be lowered, even in carriers with mild phenotypes.

In addition, further clinical evaluations should carefully focus on HF management. In fact, it has been reported that patients with *RBM20*-associated DCM also present an early onset of severe DCM and a high likelihood of end-stage HF and heart transplantation [32,33,34,35,36]. Unfortunately, in this family, no one lived long enough to develop HF symptoms. However, thanks to genetic screening, we identified four asymptomatic carriers with a mild DMC phenotype (Figure 1). From now on, they will all be closely followed up and treated according to the ESC HF guidelines [12]. In this regard, the LVEF of patient III.7 has already dropped in the most recent TTE. Moreover, AF was also a common arrythmia in this family. In fact, all identified carries older than 35 years old had documented AF.

Apart from that, it has been suggested that the variation in the aggressiveness of the disease can be sex-dependent [36]. It has been proposed that male carriers experience an earlier onset and a more severe disease progression [37]. Furthermore, it has been reported that 60% of all male *RBM20* carriers experienced a major cardiovascular event before the age of 40 years, while this occurred in less than 5% of females [43]. Apparently, this is not consistent with a disease exhibiting an autosomal dominant inheritance pattern. In this regard, in the present family, all individuals who suffered SD and confirmed carriers were men. However, larger studies are needed to draw more definitive conclusions.

Moreover, this work aims to highlight the inestimable value of the anatomopathological and forensic work. They are the key to identifying certain life-threatening conditions in cardiac autopsies, especially in the young [4]. These results should encourage others to establish local SCD protocols for investigating concealed inherited cardiac conditions in young patients presenting with SCD. The addition of genetic testing to autopsy investigation has proven to substantially increase the identification of SCD causes among children and young adults [1]. For instance, in a cohort of 490 young patients with SCD, 16% of inherited cardiomyopathies were identified thanks to autopsy [1]. Moreover, as already discussed, some pathogenic variants in *LMNA*, *FLNC* and *RBM20*, as seen in the presented family, have been reported in highly arrhythmogenic phenotypes with minimal to subtle structural defects [14]. Therefore, combining clinical and molecular autopsies can help to identify the underling inherited cardiomyopathy beyond SCD, even during the early stages of the cardiomyopathy [1,2,44,45]. However, despite it being strongly recommended in current guidelines [44,46,47], achieving both cardiac histopathological examination and genetic testing systematically in all young SCDs remains a significant challenge. The legal authorization mechanisms for this purpose varies among countries [48,49]. We strongly believe that when SCD in the young is suspected to be due to an arrhythmogenic inherited cardiac disease, clinical and molecular autopsies should be considered a public health priority [50]. In the family presented in this paper, post-mortem genetic testing provided an indispensable approach to uncover the genetic cause of the disease. Thanks to the forensic and genetic work, we were able to perform cascade genetic testing and provide personalized preventive measures to all relatives at risk. These data illustrate and reinforce the importance of establishing multidisciplinary working teams, including cardiac pathologists, forensic experts, specialized cardiologists and geneticists [2].

## 5. Limitations

Some relatives refused to undergo clinical or genetic testing. Some relatives who suffered sudden death could not be neither phenotypically nor genotypically evaluated. In the two obligate carriers who suffered sudden death, their positive phenotype can only be suspected. Moreover, we cannot rule out the presence of unknown genetic modifiers and environmental factors that could have affected the phenotype. Increasing the follow-up duration of the patients implanted with ICD could shed some light on the occurrence of ventricular arrhythmias.

## 6. Conclusions

We report a family with startling rates of sudden death in which a forensic and molecular autopsy provided crucial information to identify the underlying inherited cardiac disease. The pathogenic *RBM20* p.Arg636Cys variant identified in this family may be associated with a high risk of SCD, even in the presence of mild DCM phenotypes with normal LVEF. As a result, ICD implantation to prevent SCD should be carefully evaluated in all *RBM20* p.Arg636Cys carriers. Moreover, the frequent development of AF and HF progression requires specific awareness.

## Figures and Tables

**Table 1:** Summary of relatives who suffered sudden death.

Patient	Age at Death	Sudden Death Details	Prior CV History	Clinical/Genetic Status
II.3	57 years old	Unclear	Unknown	Obligate *RBM20* p.Arg636Cys carrier
II.4	37 years old	At rest (sleeping)	Unknown	Unknown
II.5	18 years old	Unclear	Unknown	Unknown
II.6	54 years old	At rest (sleeping)	Unknown	Unknown
II.8	54 years old	Unclear	Unknown	Obligate *RBM20* p.Arg636Cys carrier
III.1	44 years old	At rest (sleeping)	Unknown	Unknown
III.6	37 years old	At work	Syncope in the context of rapid atrial fibrillation	Mild DCM phenotype *RBM20* p.Arg636Cys carrier

CV: cardiovascular; DCM: dilated cardiomyopathy.

**Table 2:** Cariological phenotype evaluation of *RBM20* p.Arg636Cys carriers.

Patient (Birth Date)	Clinical Data	Basal ECG	Basal TTE	CMRI/Autopsy	Registered Arrhythmias
III.5 (9/5/1973)	Asymptomatic	Repolarization changes	Normal LVEF (60%) LV: mildly dilated	LVEF: 52% LV: mild dilated	AF
Proband III.6 (19/6/80)	SCD	Normal	Normal LVEF (60%) LV: mildly dilated	Autopsy: LV: mildly dilated	AF SCD (suspected unregistered ventricular malignant arrhythmia)
III.7 (27/6/1976)	Asymptomatic	Normal	LVEF: 52% LV: mildly dilated	LVEF: 48% LV: severely dilated	NSVT AF
III.8 (5/12/2004)	Asymptomatic	Normal	Normal LVEF (64%) LV: mildly dilated	LVEF: 51% LV: severely dilated	NSVT SVT
II.10 (20/4/1995)	Asymptomatic Surgically corrected atrial septal defect	Normal	LVEF: 56% LV: non-dilated	LVEF: 50% LV: severely dilated	None

ECGs: electrocardiogram; TTE: transthoracic echocardiogram (TTE); CMRI: cardiac magnetic resonance; SCD: sudden cardiac death; LV: left ventricle; LVEF: left ventricular ejection fraction; AF: atrial fibrillation; NSVT: Non-sustained ventricular tachycardia; SVT: Supraventricular tachycardia.

**Table 3:** Summary of all *RBM20* p.Arg636Cys carriers reported in the literature [34].

Patient	Age at Evaluation (Age of Death)	Genetic Status	Clinical Data at Diagnosis	Most Relevant Reported ECG Data	Reported LVEF
II-2 (Proband)	43 (64)	Carrier	Diagnosed thanks to family screening, asymptomatic with subclinical DCM. Evolved to syncope, HF, ICD, LVAD	AF, VT, VF	10%
II-4 (sister)	24 (25)	Unknown	Prior history of peripartum cardiomyopathy	-	-
III-1 (son)	23 (-)	Carrier	Diagnosed thanks to family screening, asymptomatic with subclinical DCM.	IVCD, Nonspecific ST-T changes	35%
III-2 (nephew)	16 (16)	Unknown	HF, heart transplant	-	21%
III-3 (nephew)	NA (14)	Unknown	HF	-	31%

AF: atrial fibrillation; DCM: dilated cardiomyopathy; HF: heart failure; ICD: implantable cardiac defibrillator; IVCD: intraventricular conduction delay; LVAD: left ventricular assistance device; LVEF: left ventricular ejection fraction; VF: ventricular fibrillation; VT: ventricular tachycardia.

**Table 4:** Single nucleotide variants reported in the ClinVar database affecting the same codon 636 *RBM20* gene (NM_001134363.3) (www.ncbi.nlm.nih.gov/clinvar/intro (accessed on 1 January 2025)).

Gene	c.DNA	Protein Change	Allele Frequency (gnomAD)	Interpretation	Submissions
*RBM20*	c.1906C>T	p.Arg636Cys (R636C)	0.00001	Conflicting interpretations of pathogenicity	4 P 1 VUS
*RBM20*	c.1906C>A	p.Arg636Ser (R636S)	Absent	P/LP	5 P 1 LP
*RBM20*	c.1907G>A	p.Arg636His (R636H)	0.00001	P/LP	13 P 2 LP
*RBM20*	c.1907G>T	p.Arg636Leu (R6363Leu)	Absent	P/LP	1 P 1 LP
*RBM20*	c.1908T>C	p.Arg636=	Absent	VUS	1 VUS

P: pathogenic, LP: likely pathogenic; VUS: variant of unknown significance; gnomAD: the Genome Aggregation Database.

## Data Availability

Data supporting this study are included within the article and/or supporting materials. Additional data are available from the corresponding authors upon reasonable request.

## Supplementary Materials

The following supporting information can be downloaded at the following website: https://www.mdpi.com/article/10.3390/jcm14030743/s1, Figure S1: Cardiac magnetic resonance images from *RMB20* p.Arg636Cys carriers with subclinical dilated cardiomyopathy, without late gadolinium enhancement.

## References

[1] Bagnall R.D., Weintraub R.G., Ingles J., Duflou J., Yeates L., Lam L., Davis A.M., Thompson T., Connell V., Wallace J. (2016). A Prospective Study of Sudden Cardiac Death among Children and Young Adults. N. Engl. J. Med..

[2] Martínez-Barrios E., Grassi S., Brión M., Toro R., Cesar S., Cruzalegui J., Coll M., Alcalde M., Brugada R., Greco A. (2023). Molecular autopsy: Twenty years of post-mortem diagnosis in sudden cardiac death. Front. Med..

[3] Votýpka P., Krebsová A., Norambuena-Poustková P., Peldová P., Pohlová Kučerová Š., Kulvajtová M., Dohnalová P., Bílek M., Stufka V., Rücklová K. (2023). Post-mortem genetic testing in sudden cardiac death and genetic screening of relatives at risk: Lessons learned from a Czech pilot multidisciplinary study. Int. J. Legal Med..

[4] Sheppard M.N., van der Wal A.C., Banner J., d’Amati G., De Gaspari M., De Gouveia R., Di Gioia C., Giordano C., Larsen M.K., Lynch M.J. (2023). Genetically determined cardiomyopathies at autopsy: The pivotal role of the pathologist in establishing the diagnosis and guiding family screening. Virchows Arch..

[5] Isbister J.C., Nowak N., Yeates L., Singer E.S., Sy R.W., Ingles J., Raju H., Bagnall R.D., Semsarian C. (2022). Concealed Cardiomyopathy in Autopsy-Inconclusive Cases of Sudden Cardiac Death and Implications for Families. J. Am. Coll. Cardiol..

[6] Isbister J.C., Nowak N., Butters A., Yeates L., Gray B., Sy R.W., Ingles J., Bagnall R.D., Semsarian C. (2021). “Concealed cardiomyopathy” as a cause of previously unexplained sudden cardiac arrest. Int. J. Cardiol..

[7] Pinto Y.M., Elliott P.M., Arbustini E., Adler Y., Anastasakis A., Böhm M., Duboc D., Gimeno J., de Groote P., Imazio M. (2016). Proposal for a revised definition of dilated cardiomyopathy, hypokinetic non-dilated cardiomyopathy, and its implications for clinical practice: A position statement of the ESC working group on myocardial and pericardial diseases. Eur. Heart J..

[8] Priori S.G., Blomström-Lundqvist C., Mazzanti A., Blom N., Borggrefe M., Camm J., Elliott P.M., Fitzsimons D., Hatala R., Hindricks G. (2015). 2015 ESC Guidelines for the management of patients with ventricular arrhythmias and the prevention of sudden cardiac death: The Task Force for the Management of Patients with Ventricular Arrhythmias and the Prevention of Sudden Cardiac Death of the European Society of Cardiology (ESC). Endorsed by: Association for European Paediatric and Congenital Cardiology (AEPC). Eur. Heart J..

[9] Reichart D., Magnussen C., Zeller T., Blankenberg S. (2019). Dilated cardiomyopathy: From epidemiologic to genetic phenotypes: A translational review of current literature. J. Intern. Med..

[10] Shoureshi P., Tan A.Y., Koneru J., Ellenbogen K.A., Kaszala K., Huizar J.F. (2024). Arrhythmia-Induced Cardiomyopathy: JACC State-of-the-Art Review. J. Am. Coll. Cardiol..

[11] Zeppenfeld K., Tfelt-Hansen J., de Riva M., Winkel B.G., Behr E.R., Blom N.A., Charron P., Corrado D., Dagres N., de Chillou C. (2022). 2022 ESC Guidelines for the management of patients with ventricular arrhythmias and the prevention of sudden cardiac death. Eur. Heart J..

[12] McDonagh T.A., Metra M., Adamo M., Gardner R.S., Baumbach A., Böhm M., Burri H., Butler J., Čelutkienė J., Chioncel O. (2021). 2021 ESC Guidelines for the diagnosis and treatment of acute and chronic heart failure. Eur. Heart J..

[13] Køber L., Thune J.J., Nielsen J.C., Haarbo J., Videbæk L., Korup E., Jensen G., Hildebrandt P., Steffensen F.H., Bruun N.E. (2016). Defibrillator Implantation in Patients with Nonischemic Systolic Heart Failure. N. Engl. J. Med..

[14] Peters S., Kumar S., Elliott P., Kalman J.M., Fatkin D. (2019). Arrhythmic Genotypes in Familial Dilated Cardiomyopathy: Implications for Genetic Testing and Clinical Management. Heart Lung Circ..

[15] Escobar-Lopez L., Ochoa J.P., Mirelis J.G., Espinosa M.Á., Navarro M., Gallego-Delgado M., Barriales-Villa R., Robles-Mezcua A., Basurte-Elorz M.T., Gutiérrez García-Moreno L. (2021). Association of Genetic Variants With Outcomes in Patients With Nonischemic Dilated Cardiomyopathy. J. Am. Coll. Cardiol..

[16] Arbelo E., Protonotarios A., Gimeno J.R., Arbustini E., Barriales-Villa R., Basso C., Bezzina C.R., Biagini E., Blom N.A., de Boer R.A. (2023). 2023 ESC Guidelines for the management of cardiomyopathies. Eur. Heart J..

[17] Brodehl A., Dieding M., Klauke B., Dec E., Madaan S., Huang T., Gargus J., Fatima A., Saric T., Cakar H. (2013). The novel desmin mutant p.A120D impairs filament formation, prevents intercalated disk localization, and causes sudden cardiac death. Circ. Cardiovasc. Genet..

[18] Wahbi K., Ben Yaou R., Gandjbakhch E., Anselme F., Gossios T., Lakdawala N.K., Stalens C., Sacher F., Babuty D., Trochu J.-N. (2019). Development and Validation of a New Risk Prediction Score for Life-Threatening Ventricular Tachyarrhythmias in Laminopathies. Circulation.

[19] Verstraelen T.E., van Lint F.H.M., Bosman L.P., de Brouwer R., Proost V.M., Abeln B.G.S., Taha K., Zwinderman A.H., Dickhoff C., Oomen T. (2021). Prediction of ventricular arrhythmia in phospholamban p.Arg14del mutation carriers-reaching the frontiers of individual risk prediction. Eur. Heart J..

[20] Sorella A., Galanti K., Iezzi L., Gallina S., Mohammed S.F., Sekhri N., Akhtar M.M., Prasad S.K., Chahal C.A.A., Ricci F. (2024). Diagnosis and management of dilated cardiomyopathy: A systematic review of clinical practice guidelines and recommendations. Eur. Heart J. Qual. Care Clin. Outcomes.

[21] Miller S.A., Dykes D.D., Polesky H.F. (1988). A simple salting out procedure for extracting DNA from human nucleated cells. Nucl. Acids Res..

[22] Gómez J., Reguero J.R., Morís C., Martín M., Alvarez V., Alonso B., Iglesias S., Coto E. (2014). Mutation Analysis of the Main Hypertrophic Cardiomyopathy Genes Using Multiplex Amplification and Semiconductor Next-Generation Sequencing. Circ. J..

[23] Gómez J., Lorca R., Reguero J.R., Morís C., Martín M., Tranche S., Alonso B., Iglesias S., Alvarez V., Díaz-Molina B. (2017). Screening of the *Filamin C* Gene in a Large Cohort of Hypertrophic Cardiomyopathy Patients. Circ. Cardiovasc. Genet..

[24] Lorca R., Aparicio A., Cuesta-Llavona E., Pascual I., Junco A., Hevia S., Villazón F., Hernandez-Vaquero D., Rodríguez Reguero J.J., Moris C. (2020). Familial Hypercholesterolemia in Premature Acute Coronary Syndrome. Insights from CholeSTEMI Registry. J. Clin. Med..

[25] Lorca R., Junco-Vicente A., Pérez-Pérez A., Pascual I., Persia-Paulino Y.R., González-Urbistondo F., Cuesta-Llavona E., Fernández-Barrio B.C., Morís C., Rubín J.M. (2022). KCNH2 p.Gly262AlafsTer98: A New Threatening Variant Associated with Long QT Syndrome in a Spanish Cohort. Life.

[26] Lorca R., Martín M., Pascual I., Astudillo A., Díaz Molina B., Cigarrán H., Cuesta-Llavona E., Avanzas P., Rodríguez Reguero J.J., Coto E. (2020). Characterization of Left Ventricular Non-Compaction Cardiomyopathy. J. Clin. Med..

[27] Lorca R., Gómez J., Martín M., Cabanillas R., Calvo J., León V., Pascual I., Morís C., Coto E., Reguero J.J.R. (2019). Insights Into Hypertrophic Cardiomyopathy Evaluation Through Follow-up of a Founder Pathogenic Variant. Rev. Española Cardiol. (Engl. Ed.).

[28] Richards S., Aziz N., Bale S., Bick D., Das S., Gastier-Foster J., Grody W.W., Hegde M., Lyon E., Spector E. (2015). Standards and guidelines for the interpretation of sequence variants: A joint consensus recommendation of the American College of Medical Genetics and Genomics and the Association for Molecular Pathology. Genet. Med..

[29] Salgado M., Gonzalez-Urbistondo F., Aparicio A., Cachero A., Helguera C., Lorca R. (2023). Genetic cause of sudden cardiac death. Abstracts of the Heart Failure 2023 and the World Congress on Acute Heart Failure, 20–23 May 2023, Prague, Czechia. Eur. J. Heart Fail..

[30] Yafasova A., Doi S.N., Thune J.J., Nielsen J.C., Haarbo J., Bruun N.E., Gustafsson F., Eiskjær H., Hassager C., Svendsen J.H. (2024). Effect of Implantable Cardioverter-defibrillators in Nonischemic Heart Failure According to Background Medical Therapy: Extended Follow-up of the DANISH Trial. J. Card. Fail..

[31] Stecker E.C., Vickers C., Waltz J., Socoteanu C., John B.T., Mariani R., McAnulty J.H., Gunson K., Jui J., Chugh S.S. (2006). Population-Based Analysis of Sudden Cardiac Death With and Without Left Ventricular Systolic Dysfunction. J. Am. Coll. Cardiol..

[32] Beqqali A., Bollen I.A.E., Rasmussen T.B., Van Den Hoogenhof M.M., Van Deutekom H.W.M., Schafer S., Haas J., Meder B., Sørensen K.E., Van Oort R.J. (2016). A mutation in the glutamate-rich region of RNA-binding motif protein 20 causes dilated cardiomyopathy through missplicing of titin and impaired Frank–Starling mechanism. Cardiovasc. Res..

[33] Brauch K.M., Karst M.L., Herron K.J., de Andrade M., Pellikka P.A., Rodeheffer R.J., Michels V.V., Olson T.M. (2009). Mutations in ribonucleic acid binding protein gene cause familial dilated cardiomyopathy. J. Am. Coll. Cardiol..

[34] Li D., Morales A., Gonzalez-Quintana J., Norton N., Siegfried J.D., Hofmeyer M., Hershberger R.E. (2010). Identification of Novel Mutations in RBM20 in Patients with Dilated Cardiomyopathy. Clin. Transl. Sci..

[35] Refaat M.M., Lubitz S.A., Makino S., Islam Z., Frangiskakis J.M., Mehdi H., Gutmann R., Zhang M.L., Bloom H.L., MacRae C.A. (2012). Genetic variation in the alternative splicing regulator RBM20 is associated with dilated cardiomyopathy. Heart Rhythm..

[36] Cannie D.E., Protonotarios A., Bakalakos A., Syrris P., Lorenzini M., De Stavola B., Bjerregaard L., Dybro A.M., Hey T.M., Hansen F.G. (2023). Risks of Ventricular Arrhythmia and Heart Failure in Carriers of *RBM20* Variants. Circ Genom. Precis. Med..

[37] Jordà P., Toro R., Diez C., Salazar-Mendiguchía J., Fernandez-Falgueras A., Perez-Serra A., Coll M., Puigmulé M., Arbelo E., García-Álvarez A. (2021). Malignant Arrhythmogenic Role Associated with RBM20: A Comprehensive Interpretation Focused on a Personalized Approach. J. Pers. Med..

[38] Gaertner A., Klauke B., Felski E., Kassner A., Brodehl A., Gerdes D., Stanasiuk C., Ebbinghaus H., Schulz U., Dubowy K.-O. (2020). Cardiomyopathy-associated mutations in the RS domain affect nuclear localization of RBM20. Hum. Mutat..

[39] Wells Q.S., Becker J.R., Su Y.R., Mosley J.D., Weeke P., D’Aoust L., Ausborn N.L., Ramirez A.H., Pfotenhauer J.P., Naftilan A.J. (2013). Whole exome sequencing identifies a causal RBM20 mutation in a large pedigree with familial dilated cardiomyopathy. Circ. Cardiovasc. Genet..

[40] Chami N., Tadros R., Lemarbre F., Lo K.S., Beaudoin M., Robb L., Labuda D., Tardif J.-C., Racine N., Talajic M. (2014). Nonsense mutations in BAG3 are associated with early-onset dilated cardiomyopathy in French Canadians. Can. J. Cardiol..

[41] Brodehl A., Ebbinghaus H., Gaertner-Rommel A., Stanasiuk C., Klauke B., Milting H. (2019). Functional analysis of DES-p.L398P and RBM20-p.R636C. Genet. Med..

[42] Wilsbacher L.D. (2020). Clinical Implications of the Genetic Architecture of Dilated Cardiomyopathy. Curr. Cardiol. Rep..

[43] Hey T.M., Rasmussen T.B., Madsen T., Aagaard M.M., Harbo M., Mølgaard H., Møller J.E., Eiskjær H., Mogensen J. (2019). Pathogenic RBM20-Variants Are Associated With a Severe Disease Expression in Male Patients With Dilated Cardiomyopathy. Circ. Heart Fail..

[44] Wilde A.A.M., Semsarian C., Márquez M.F., Shamloo A.S., Ackerman M.J., Ashley E.A., Sternick E.B., Barajas-Martinez H., Behr E.R., Bezzina C.R. (2022). European Heart Rhythm Association (EHRA)/Heart Rhythm Society (HRS)/Asia Pacific Heart Rhythm Society (APHRS)/Latin American Heart Rhythm Society (LAHRS) Expert Consensus Statement on the state of genetic testing for cardiac diseases. Europace.

[45] Neves R., Tester D.J., Simpson M.A., Behr E.R., Ackerman M.J., Giudicessi J.R. (2022). Exome Sequencing Highlights a Potential Role for Concealed Cardiomyopathies in Youthful Sudden Cardiac Death. Circ. Genom. Precis. Med..

[46] Stiles M.K., Wilde A.A.M., Abrams D.J., Ackerman M.J., Albert C.M., Behr E.R., Chugh S.S., Cornel M.C., Gardner K., Ingles J. (2021). 2020 APHRS/HRS expert consensus statement on the investigation of decedents with sudden unexplained death and patients with sudden cardiac arrest, and of their families. Heart Rhythm.

[47] Kelly K.L., Lin P.T., Basso C., Bois M., Buja L.M., Cohle S.D., d’Amati G., Duncanson E., Fallon J.T., Firchau D. (2023). Sudden cardiac death in the young: A consensus statement on recommended practices for cardiac examination by pathologists from the Society for Cardiovascular Pathology. Cardiovasc. Pathol..

[48] Grassi S., Campuzano O., Coll M., Brión M., Arena V., Iglesias A., Carracedo Á., Brugada R., Oliva A. (2020). Genetic variants of uncertain significance: How to match scientific rigour and standard of proof in sudden cardiac death?. Legal Med..

[49] Basso C., Aguilera B., Banner J., Cohle S., d’Amati G., De Gouveia R.H., Di Gioia C., Fabre A., Gallagher P.J., Leone O. (2017). Guidelines for autopsy investigation of sudden cardiac death: 2017 update from the Association for European Cardiovascular Pathology. Virchows Arch..

[50] Grassi S., Campuzano O., Coll M., Cazzato F., Sarquella-Brugada G., Rossi R., Arena V., Brugada J., Brugada R., Oliva A. (2021). Update on the Diagnostic Pitfalls of Autopsy and Post-Mortem Genetic Testing in Cardiomyopathies. Int. J. Mol. Sci..

