# Supplementary material for: RBM20 p.Arg636Cys: A Pathogenic Variant Identified in a Family with Several Cases of Unexpected Sudden Deaths
Source: J Clin Med. 2025 Jan 24;14(3):743. doi: 10.3390/jcm14030743 (PMC11818836; doi:10.3390/jcm14030743)
Supplement: Supplementary file 1 [file jcm-14-00743-s001.zip › jcm-3428052-supplementary.pdf]

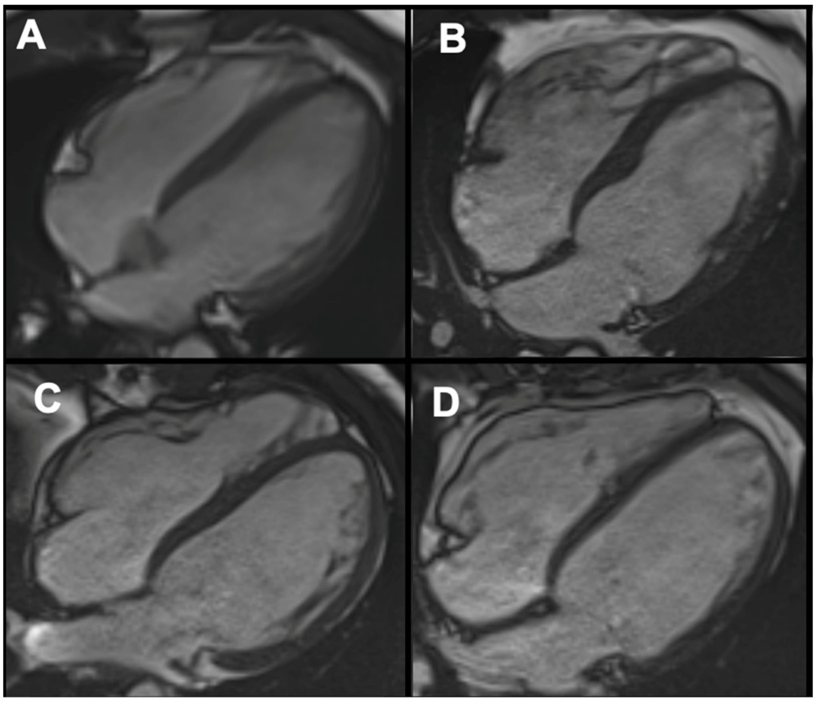

Figure S1. Cardiac magnetic resonance images from *RMB20* p.Arg636Cys carriers with subclinical dilated cardiomyopathy, without late gadolinium enhancement S1A) Patient III.5; S1B) Patient III.7; S1C) Patient III.10; S1D) Patient III.8.
